# Safety of an SV-1 Cell Line-Based Varicella Vaccine Before and After Integration into the Expanded Program on Immunization: A Real-World Study in Jiangsu Province, China

**DOI:** 10.3390/vaccines14030200

**Published:** 2026-02-24

**Authors:** Jing Yu, Yurong Li, Zhiguo Wang, Xiang Sun, Guodong Kang, Borong Xu, Yuanyuan Zhu, Xun Li, Xiaozhe Song, Yonghong Sun, Dongsheng Liu, Yuan Ren, Xueyan Sha, Ran Hu

**Affiliations:** 1 Department of Immunization Program, Jiangsu Provincial Center for Disease Control and Prevention, Nanjing 210009, China; yujing@jscdc.cn (J.Y.); njwang1718@163.com (Z.W.); sunstone1@163.com (X.S.); kanggd@jscdc.cn (G.K.); jscdcxbr@163.com (B.X.); zyy2018@foxmail.com (Y.Z.); 18020183211@163.com (X.L.); 2 Sinovac Biotech Co., Ltd., Beijing 100085, China; liyr8134@sinovac.com (Y.L.); renyuan@sinovac.com (Y.R.); 3 School of Public Health, Nanjing Medical University, Nanjing 211166, China; 4 Department of Immunization Program, Xuzhou Center for Disease Control and Prevention, Xuzhou 221018, China; xzcdpc@126.com (X.S.); sunyonghong2528@163.com (Y.S.); 15895216226@163.com (D.L.); 5 Sinovac (Dalian) Vaccine Technology Co., Ltd., Dalian 116100, China; shaxy@sinovac.com

**Keywords:** varicella vaccine, SV-1 cell line, adverse events following immunization, vaccine safety, children, vaccination, adverse events

## Abstract

**Background/Objectives**: Varicella is a highly contagious childhood disease that may cause severe complications in susceptible populations. The SV-1 cell line-based varicella vaccine (VarV [SV-1]) has been increasingly used in routine immunization; however, safety and reporting patterns during the transition from partial use to full Expanded Program on Immunization (EPI) implementation remain poorly characterized. This study aimed to evaluate the safety profile and reporting dynamics of VarV (SV-1) before and after its incorporation into the Expanded Program on Immunization in Jiangsu Province, China. **Methods**: A retrospective observational study was conducted using data from the Jiangsu Provincial Immunization Integrated Service Management Information System and the Chinese National Adverse Event Following Immunization (AEFI) Information System (CNAEFIS), including all reported AEFI following VarV (SV-1) vaccination among children under 6 years of age during 2021–2023. Temporal trends, distribution characteristics, and factors associated with AEFI reporting were assessed using descriptive analyses, negative binomial (NB) regression models, and interrupted time series (ITS) analysis. **Results**: A total of 1,208,500 doses of VarV (SV-1) were administered, and 634 AEFI cases were reported, corresponding to an overall reporting rate of 52.46 per 100,000 doses. Most reported events were mild, self-limiting common reactions, predominantly pyrexia and injection-site reactions. No serious adverse events were identified. Although no immediate level change was observed at EPI implementation, a significant increasing post-EPI trend was detected, consistent with enhanced surveillance sensitivity rather than a change in intrinsic vaccine safety. Abnormal reactions were rare and resolved without sequelae. **Conclusions**: AEFI reporting rates following VarV (SV-1) vaccination in Jiangsu Province were within expected ranges, predominantly mild and reversible. Findings support the favorable safety profile of the VarV (SV-1) in routine childhood immunization programs and provide real-world evidence to support continued implementation of the two-dose varicella vaccination strategy within the EPI.

## 1. Introduction

Varicella is an acute infectious disease caused by the varicella-zoster virus (VZV) and predominantly affects children. It is characterized by high transmissibility and marked seasonality. Although most cases are self-limiting, varicella can lead to severe complications such as secondary bacterial skin infections, pneumonia, and encephalitis, particularly among immunocompromised individuals, thereby posing a considerable burden to public health systems [1].

Vaccination with the live attenuated varicella vaccine (VarV) is widely recognized as the most effective and cost-efficient strategy for the prevention and control of varicella. The World Health Organization (WHO) recommends the use of varicella vaccines to prevent varicella in children in settings where the disease represents an important public health problem. Given the higher effectiveness of a two-dose schedule, WHO recommends preferential implementation of two doses, as one-dose programs are less effective in preventing sustained virus circulation and outbreaks [1]. Evidence from multiple settings has demonstrated that two-dose VarV schedules substantially reduce varicella incidence, breakthrough infections, and outbreak occurrence compared with single-dose strategies [1,2,3].

In China, varicella vaccination was initially implemented outside the national EPI, resulting in heterogeneous coverage across regions and persistent outbreaks. To strengthen varicella control, several provinces and municipalities gradually piloted the inclusion of VarV into locally funded immunization programs. In Jiangsu Province, VarV was first introduced through city-level pilot programs beginning in 2018 and was officially incorporated into the provincial EPI on 1 January 2023, with a standardized two-dose schedule provided free of charge to eligible children under 6 years of age [4]. The period from 2021 to 2022 represented a transitional phase characterized by partial implementation, whereas from 2023 onward, VarV was uniformly included across the entire province.

From a global perspective, WHO emphasizes that the successful introduction of a new vaccine into routine immunization programs should be accompanied by strengthened post-marketing safety surveillance, particularly during periods of rapid scale-up in coverage [5]. In passive surveillance systems, increases in reported AEFI after programmatic changes are frequently attributable to improved reporting sensitivity, enhanced provider awareness, and increased public attention, rather than due to the true increases in vaccine-related risk [6]. Distinguishing such surveillance artifacts from genuine safety signals is therefore a central objective of post-introduction vaccine safety evaluations.

The varicella vaccine based on the SV-1 cell line represents a domestically developed product in China. The SV-1 (formerly SLF-1) human embryonic lung fibroblast diploid cell strain was independently developed by Sinovac (Dalian) Vaccine Technology Co., Ltd. (hereafter Sinovac), in Dalian, China, and constitutes a new-generation human diploid cell substrate [7]. Since 2020, Sinovcac’s VarV (SV-1) has been progressively introduced into routine immunization services in Jiangsu Province, with approximately 3 million doses administered. Following its incorporation into the provincial Expanded Program on Immunization (EPI), both the number of administered doses (approximately 16 million) and the geographic coverage (approximately 2700 counties/districts) of VarV (SV-1) increased substantially. This large-scale rollout provided a valuable opportunity to evaluate the vaccine’s safety profile under real-world, programmatic conditions.

Despite the widespread use of varicella vaccines globally, WHO has noted that evidence on vaccine safety should be continuously generated and updated using real-world data, because post-licensure safety monitoring and real-world evidence are critical components of global vaccine safety assessment and policy guidance [6]. In China, although several surveillance studies have assessed AEFI following varicella vaccination, systematic evaluations conducted during periods of rapid EPI expansion remain limited, especially at the provincial level.

Therefore, using data from the Jiangsu Provincial Immunization Integrated Service Management Information System and the national AEFI surveillance system, this study aimed to evaluate the safety profile of VarV (SV-1) among children under 6 years of age—the age group eligible for varicella vaccination in Jiangsu Province—during 2021–2023. Findings from this study are intended to contribute real-world evidence to support ongoing vaccine safety monitoring, inform immunization policy optimization, and strengthen public confidence in routine childhood vaccination programs.

## 2. Materials and Methods

### 2.1. Study Design and Data Sources

This was a retrospective, real-world observational study based on passive surveillance data of AEFI. Data on suspected AEFI cases were obtained from the national AEFI Surveillance System, a component of the Chinese Center for Disease Control and Prevention information platform, which collects reports from all vaccination providers nationwide.

Information on vaccination history, including administered doses, vaccination dates, vaccine type, and demographic characteristics, was retrieved from the Jiangsu Provincial Immunization Integrated Service Management Information System. The study included all reported AEFI cases following administration of the VarV (SV-1) among children under 6 years of age in Jiangsu Province during the period from 1 January 2021 to 31 December 2023.

As the study involved secondary analysis of de-identified surveillance data collected for routine public health purposes, ethical review and informed consent were waived.

### 2.2. Study Population and Vaccination Schedule

According to the Implementation Plan for Incorporating the Live Attenuated Varicella Vaccine into the Childhood Immunization Program of Jiangsu Province [4], the study population comprised children born on or after 1 January 2017, who were registered in the provincial immunization information system, had resided in Jiangsu Province for at least 2 months, and completed the scheduled VarV (SV-1) vaccination regimen.

Sinovac (Dalian) live attenuated varicella vaccine (Oka strain) was supplied as a lyophilized product, stored at 2–8 °C, reconstituted to 0.5 mL shortly before use, and administered subcutaneously in the deltoid region/upper arm. The recommended vaccination schedule consisted of two doses: the first dose administered at 12–18 months of age, and the second dose administered at least 3 months after the first dose and typically before 4 years of age.

### 2.3. Definitions and Classification of AEFI

AEFI reporting, investigation, and diagnosis were conducted in accordance with the National Surveillance Protocol for Suspected Adverse Events Following Immunization [8], which is aligned with WHO guidelines [6]. Based on etiological assessment, AEFI were classified into the following categories: (1) adverse reactions, including common reactions and abnormal reactions (both confirmed and suspected vaccine-related reactions); (2) psychogenic reactions; (3) vaccine quality incidents; (4) vaccination errors; and (5) coincidental events. Cases classified as suspected abnormal reactions were included in the analysis of abnormal reactions.

In addition, all reported adverse reactions were further coded using the Medical Dictionary for Regulatory Activities (MedDRA V27), with Preferred Terms (PTs) used for classification of clinical diagnoses and time-to-onset analyses.

### 2.4. Statistical Analysis

All statistical analyses were conducted in accordance with the characteristics of passive AEFI surveillance data, using administered vaccine doses as denominators.

#### 2.4.1. Descriptive Analyses and Crude Reporting Rates

Vaccination dose counts and AEFI case data were extracted from the provincial immunization information system and the national AEFI surveillance system. Data cleaning and aggregation were performed using Microsoft Excel 2019, and all statistical analyses were conducted using R software (version 4.5.2).

AEFI reporting rates were calculated as the number of reported AEFI cases per 100,000 administered doses. Exact Poisson methods were used to calculate 95% confidence intervals (95% CIs). Reporting rates were descriptively summarized by sex, age group (1–6 years), calendar year (2021–2023), calendar quarter, dose number (first or second dose), geographic region (southern, central, and northern Jiangsu), city.

Differences in crude reporting rates across strata were evaluated using contingency-table methods. Pearson’s chi-square test was used when expected cell counts were sufficient; otherwise, Fisher’s exact test with Monte Carlo simulation was applied. For ordered categorical variables, such as age group, Cochran–Armitage trend tests were performed. *p*-values from multiple comparisons were adjusted using the Benjamini–Hochberg (BH) false discovery rate (FDR) method.

#### 2.4.2. Temporal Trend and Annual Comparison Analyses

Temporal trends in AEFI reporting rates over calendar years were assessed using Poisson regression models for rate-based temporal comparisons, with calendar year specified either as a continuous variable (to evaluate overall trends) or as a categorical variable (to enable year-to-year comparisons). The logarithm of administered doses was included as an offset term to model reporting rates rather than absolute counts.

For selected analyses, data from 2021 to 2022 were combined to represent the pre–EPI period and compared with data from 2023, when VarV was incorporated into the provincial EPI across Jiangsu Province. Rate ratios (RRs) and corresponding 95% CIs were estimated using exact Poisson methods where appropriate.

#### 2.4.3. Multivariable Regression Analyses

Multivariable analyses were performed using count regression models to examine factors associated with AEFI reporting rates. The number of reported AEFI cases was specified as the dependent variable, with the logarithm of administered vaccine doses included as an offset term.

NB mixed-effects regression models with city-level random intercepts were selected a priori to account for overdispersion and heterogeneity in reporting practices across geographic areas inherent to passive surveillance data. The model was specified such that the number of reported AEFI cases was modeled as a function of sex, age, dose number, and region, with a city-level random intercept and the logarithm of administered doses included as an offset term. Adjusted incidence rate ratios (aIRRs) with 95% confidence intervals (CIs) were estimated.

Model fit was evaluated using standard goodness-of-fit statistics and information criteria. Sensitivity analyses using Poisson regression models with robust (sandwich) standard errors were conducted to assess the robustness of effect estimates.

#### 2.4.4. Interrupted Time Series Analysis

To assess the potential impact of incorporating VarV into the provincial Expanded Program on Immunization, an interrupted time series (ITS) analysis was conducted using quarterly aggregated AEFI counts and administered doses from 2021 to 2023. Segmented Poisson regression models were fitted, with the logarithm of administered doses included as an offset term.

The models included terms for the underlying pre-intervention time trend, an indicator variable representing the EPI implementation period (pre-EPI: 2021–2022; post-EPI: 2023), and an interaction term capturing changes in the post-intervention trend. Robust (sandwich) standard errors were applied to mitigate potential autocorrelation and model misspecification. Results were expressed as incidence rate ratios (IRRs) with 95% CIs.

Quarterly time intervals were used as the unit of aggregation, with AEFI counts and administered doses summed for each calendar quarter.

This approach allowed differentiation between secular temporal trends and changes potentially associated with the province-wide implementation of the EPI.

#### 2.4.5. Interpretation of Estimates

All regression estimates were interpreted as reporting rate ratios rather than causal measures of vaccine-associated risk, reflecting associations observed within a passive surveillance system. All statistical tests were two-sided, with a significance level of α = 0.05.

## 3. Results

### 3.1. Basic Characteristics of Vaccination and AEFI Reports

#### 3.1.1. Vaccination Doses

During 2021–2022, a total of 213,300 doses of VarV (SV-1) were administered to children under 6 years of age in Jiangsu Province, including 135,300 first doses and 78,000 s doses. Following the incorporation of VarV into the provincial EPI on 1 January 2023, a total of 995,200 doses were administered in 2023, comprising 552,500 first doses and 442,700 s doses. By age group, the numbers of administered doses among children aged 1 to 6 years in 2023 were 168,100, 68,000, 85,900, 335,700, 252,000, and 85,500, respectively (Supplementary Table S1a).

#### 3.1.2. Overall AEFI Reporting

During 2021–2023, a total of 634 AEFI cases were reported following VarV (SV-1) vaccination among children under 6 years of age in Jiangsu Province, corresponding to an overall reporting rate of 52.46 per 100,000 administered doses (Table 1).

Year-to-year comparisons of AEFI reporting rates showed no statistically significant differences between individual calendar years. Specifically, the reporting rate in 2022 was similar to that in 2021 (RR = 1.08, 95% CI: 0.64–1.93; *p* = 0.897). Although a higher reporting rate was observed in 2023 compared with 2021 (RR = 1.40, 95% CI: 0.88–2.38), this difference did not reach statistical significance (*p* = 0.182). In contrast, when data from 2021 to 2022 were combined as the pre-EPI baseline period, the AEFI reporting rate in 2023 was significantly higher (RR = 1.31, 95% CI: 1.05–1.66; *p* = 0.016) (Table 1 and Table S1b).

### 3.2. Analysis of AEFI Reporting

#### 3.2.1. Distribution Characteristics of AEFI

Crude reporting rates differed significantly by age group and dose number (*p* < 0.05).

AEFI were most frequently reported among children aged 1 year, with a reporting rate of 79.39 per 100,000 doses, followed by a gradual decline trend with increasing age.

The reporting rate following the first dose was significantly higher than that following the second dose (57.01 vs. 46.48 per 100,000 doses, *p* < 0.05).

Regional analysis showed significant differences in AEFI reporting rates across regions (*p* = 0.015), with the highest reporting rate observed in northern Jiangsu (56.63 per 100,000 doses) and the lowest in central Jiangsu (41.57 per 100,000 doses) (Table 2 and Table S2).

#### 3.2.2. Multivariable Regression Analysis

In multivariable mixed-effects regression analyses accounting for city-level clustering, AEFI reporting rates were significantly lower among children aged 2 years and older compared with those aged 1 year, with a clear decreasing trend observed with increasing age. After adjustment for potential confounders, no statistically significant associations were observed for sex or dose number (Table 3).

Model diagnostics and comparative assessments demonstrated that a multilevel NB regression model with city-level random intercepts provided the best fit to the data, adequately accounting for overdispersion and heterogeneity in reporting across cities. No evidence of residual overdispersion or zero inflation was detected based on simulation-based diagnostics (Supplementary Table S3).

Sensitivity analyses using multivariable regression models without random effects yielded broadly consistent estimates, supporting the robustness of the primary findings (Supplementary Table S4).

#### 3.2.3. Impact of Incorporation into the Expanded Program on Immunization

Prior to incorporation into the provincial EPI, the AEFI reporting rate was 41.73 per 100,000 administered doses during 2021–2022. Following incorporation, the reporting rate was 54.76 per 100,000 in 2023 (see Supplementary Table S1a).

Interrupted time series analysis using quarterly aggregated data indicated no significant underlying temporal trend in AEFI reporting rates prior to implementation of the EPI (IRR = 1.03, 95% CI: 0.99–1.08, *p* = 0.153). No statistically significant immediate change in reporting rates was observed at the time of EPI implementation (IRR = 0.97, 95% CI: 0.87–1.08, *p* = 0.585).

However, a significant increase in the post-intervention trend was observed following EPI implementation, with AEFI reporting rates increasing by approximately 13% per quarter (IRR = 1.13, 95% CI: 1.05–1.21, *p* = 0.001).

### 3.3. Clinical Diagnosis of Adverse Events

Dose-specific analyses showed that overall AEFI reporting rates were lower after the second dose than after the first dose (46.28 vs. 56.58 per 100,000 doses; RR = 0.82, 95% CI: 0.70–0.96; *p* = 0.014), a pattern also observed for all common reactions (RR = 0.82, 95% CI: 0.69–0.96; *p* = 0.027).

Pyrexia was the most frequently reported adverse reaction and occurred significantly less often after the second dose (RR = 0.66, 95% CI: 0.55–0.81; *p* = 0.001), with consistent reductions for temperatures ≥38.6 °C (RR = 0.62, 95% CI: 0.47–0.83; *p* = 0.006) and 37.6–38.5 °C (RR = 0.68, 95% CI: 0.51–0.91; *p* = 0.019), whereas no significant difference was observed for low-grade pyrexia (37.1–37.5 °C).

In contrast, local reactions were reported more frequently after the second dose, including injection-site induration (RR = 1.96, 95% CI: 1.24–3.09; *p* = 0.010) and injection-site erythema and swelling (RR = 1.73, 95% CI: 1.24–2.40; *p* = 0.006). Large local reactions (>5.0 cm) were rare but occurred more often after the second dose for erythema and swelling (RR = 3.17, 95% CI: 1.12–9.00; *p* = 0.049).

Abnormal reactions were rare, with no significant differences in reporting rates between the first and second doses overall or across specific abnormal reaction categories (Table 4).

### 3.4. Time to Onset and Outcomes of Adverse Reactions

Most reported adverse reactions occurred on the day of vaccination or within 1–3 days after vaccination. Overall, 66.35% of AEFI occurred on the day of vaccination and 32.22% within 1–3 days, with fewer than 2% occurring 4 days or later. A similar time-to-onset pattern was observed for all common reactions, including pyrexia, injection site erythema and swelling, and injection site induration, which predominantly occurred on the day of vaccination or shortly thereafter.

Abnormal reactions were uncommon and also occurred mainly within 3 days after vaccination. Only a small proportion of abnormal reactions had an onset of 4 days or later, and no delayed-onset clustering was observed. (Table 5).

All reported abnormal reactions were mild to moderate in severity and resolved without sequelae. No serious adverse events were identified during the study period.

## 4. Discussion

This study uniquely evaluated the safety of VarV (SV-1) under a quasi-experimental setting created by EPI integration. In this retrospective, real-world study based on provincial AEFI surveillance data, we conducted a comprehensive safety evaluation of large-scale administration of the VarV (SV-1) among children under 6 years of age in Jiangsu Province during 2021–2023. The results indicate that VarV (SV-1) maintained a favorable safety profile following incorporation into the EPI. The overall AEFI reporting rate was 52.46 per 100,000 administered doses, with the majority of reported events being mild, self-limiting common reactions. No serious adverse events were identified, and all abnormal reactions resolved without sequelae. The spectrum, timing, and outcomes of reported AEFI were consistent with findings from previous domestic surveillance studies of varicella vaccines [9].

The overall AEFI reporting rate observed in this study was slightly higher than recent years national averages (26.31, 23.91, 32.33 per 100,000) [10,11,12], and was comparable to the reported AEFI rate for varicella vaccination among children aged 1–12 years in Jiangsu Province in 2022 (46.83 per 100,000) [13]. Given that varicella vaccine has not yet been included in China’s Expanded Program on Immunization (EPI), resulting in relatively low national vaccination coverage, the comparatively lower level of AEFI surveillance is understandable. It was also similar to the provincial overall vaccination AEFI reporting rate during 2021–2022 (41.73 vs. 42.45 per 100,000), while the reporting rate in 2023 was slightly lower than the overall provincial level reported in the same year (54.76 vs. 60.41 per 100,000) [14].

Compared with the reported incidence of adverse reactions (7.08%) associated with other commonly used domestic varicella vaccines [15], as well as the early post-marketing safety surveillance data (approximately three years) of internationally comparable products (67.5 per 100,000; Merck) [16], the AEFI reporting rate observed in this study was slightly lower. In contrast, the rate was marginally higher than the AEFI reporting rate observed following large-scale vaccination with this product among children aged 7–12 years in Jiangsu Province (33.4 per 100,000), which may be attributable to the higher incidence of adverse reactions in younger age groups [17,18,19]. In addition, the AEFI reporting rate was comparable to that of the live attenuated herpes zoster vaccine containing the same antigen but with different antigen content and indications (52.81 per 100,000), as reported in national surveillance data for 2023 [12].

Following the official inclusion of VarV into the provincial EPI in January 2023, the reporting rate increased significantly. Importantly, this increase is unlikely to reflect a true elevation in vaccine-related safety. Rather, it most plausibly reflects programmatic and surveillance-related factors, including expanded vaccination coverage, improved sensitivity of the AEFI reporting system, enhanced training and awareness among vaccination providers, and increased parental attention to newly introduced EPI vaccines [20].

The ITS analysis further supports this interpretation. No significant immediate change in AEFI reporting rates was observed at the time of province-wide EPI implementation, suggesting a relatively smooth transition from pilot implementation to full integration without abrupt disruptions in safety reporting. In contrast, a gradual but statistically significant increase in post-intervention reporting trends was observed. Such delayed increases are characteristic of passive surveillance systems following policy changes and are generally attributed to cumulative improvements in reporting completeness and surveillance sensitivity rather than to changes in the intrinsic safety profile of the vaccine. Similar post-implementation reporting dynamics have been documented in evaluations of other vaccines introduced into routine immunization programs, underscoring the importance of distinguishing reporting behavior from biological risk [18,20,21]

Higher AEFI reporting rates were observed among children aged 1 year, a pattern that has also been reported in other regional studies in China [13,14]. A similar trend was observed following large-scale administration of this vaccine among children aged 7–12 years in Jiangsu Province, where relatively lower AEFI reporting rates were reported in older children, suggesting a decrease in AEFI reporting with increasing age [17]. This age-related difference may be partially attributable to biological factors, as younger children may exhibit more pronounced systemic and local immune responses, potentially contributing to higher frequencies of pyrexia and injection-site reactions. In addition, heightened parental vigilance and more frequent healthcare contact during early childhood may further increase the likelihood of AEFI detection and reporting [19,22].

Marked regional differences in AEFI reporting rates were observed, with higher rates in northern Jiangsu and lower rates in central Jiangsu. These differences are likely influenced by variations in the timing of EPI implementation, local surveillance capacity, healthcare provider reporting practices, and public awareness of vaccine safety monitoring. Southern Jiangsu, where VarV had been incorporated into local government-funded immunization programs earlier, may have benefited from more established surveillance systems and reporting routines. Such regional heterogeneity is a well-recognized feature of passive surveillance systems and should be interpreted in the context of programmatic and operational differences rather than as evidence of differential vaccine safety [21,22].

Consistent with previous studies of this vaccine, most reported AEFI were common reactions, predominantly pyrexia and local injection-site reactions, typically occurring on the day of vaccination [17,23]. The majority of AEFI were reported within three days after vaccination; these events were self-limiting and resolved without medical intervention, which is consistent with provincial surveillance findings from 2017 to 2023 [14]. The lower reporting rate of pyrexia following the second dose may reflect a reduced systemic inflammatory response after immunological priming [24]. Similar to other commonly used varicella vaccines in China, passive surveillance reports were mainly characterized by mild symptoms such as fever, redness, swelling, and induration at the injection site [15,25]. Compared with post-marketing safety surveillance data reported during approximately the first three years after licensure of the Merck varicella vaccine, no reports of rash, suspected vaccine failure, herpes zoster, pharyngitis, cellulitis, hepatic disorders, pneumonia, or other commonly reported events were identified for this vaccine during the study period [16].

Abnormal reactions were rare and generally mild. Compared with other commonly used varicella vaccines in China, the reporting rate of abnormal reactions for this vaccine was relatively lower (1.66 vs. 6.76 per 100,000) [25]. In comparison with internationally used varicella vaccines, no additional serious adverse events—such as erythema multiforme, Stevens–Johnson syndrome (SJS), arthropathy, thrombocytopenia, anaphylactic shock, vasculitis, aplastic anemia, neuropathy, convulsions, ataxia, encephalopathy, or meningitis—were reported during the study period [16].

Since the pilot implementation of the varicella vaccination program in Jiangsu Province, both the overall incidence of varicella and the frequency of outbreak events have shown a significant downward trend. In 2024, the total number of reported varicella cases in the province markedly decreased compared with 2019, with particularly pronounced reductions observed among children aged 0–6 years. In addition, the number of varicella-related public health emergencies was substantially lower than that reported in 2019. These findings suggest that population-level immunity has gradually increased following the introduction of the vaccination program. Moreover, the most frequently reported adverse reactions were transient and mild, predominantly local injection-site redness and swelling (68.4%) and low-grade fever (29.1%) [26]. These trends may also reflect the real-world effectiveness of this vaccine in the local setting, in conjunction with its favorable safety profile.

Several limitations of this study should be acknowledged. First, the analysis was based on passive AEFI surveillance data, which are subject to underreporting, differential reporting practices, and variations in healthcare-seeking behavior and caregiver awareness. Second, passive surveillance systems may be less sensitive to extremely rare or delayed adverse events. Third, reporting rates reflect observed associations rather than causal relationships between vaccination and adverse events. Given the passive surveillance nature of the data, the estimated IRRs reflect associations in reporting rates rather than causal effects on true incidence. Nevertheless, the large population coverage, dose-based denominators, and use of multiple complementary analytical approaches strengthen the robustness and public health relevance of the findings. Future studies integrating active surveillance, electronic health records, and signal detection methodologies could further enhance vaccine safety evaluation.

In conclusion, the AEFI reporting rates following VarV (SV-1) vaccination among children under 6 years of age in Jiangsu Province during 2021–2023 were within expected ranges, with reported events predominantly mild and reversible. No serious adverse events were identified. Previous clinical trials and post-marketing studies have consistently demonstrated the vaccine’s favorable protective efficacy, safety profile, and immunogenicity [17,19,23,26,27,28,29,30]. The findings of this study further support the overall safety of the SV-1 cell line-based varicella vaccine when used in routine childhood immunization programs and provide empirical evidence supporting the continued implementation and expansion of the two-dose varicella vaccination strategy. Ongoing strengthening of surveillance quality and analytical capacity will remain essential to ensure vaccine safety and to inform evidence-based immunization policy.

At present, the varicella vaccine has been licensed in China for 25 years. However, it has not yet been fully incorporated into the national Expanded Program on Immunization (EPI), and most existing implementations remain regional pilot projects [31,32]. Several provinces and municipalities—including Tianjin, Shanghai, and Jiangsu—as well as Shenzhen in Guangdong Province and Qingdao and Weifang in Shandong Province have incorporated the varicella vaccine into their local immunization programs [32].

The annual number of live births and the corresponding natality rates in the region were 564,300 (6.66‰) in 2020 and 479,800 (5.65‰) in 2021. The coverage of the first dose of varicella vaccine between 2021 and 2022 was only 63.35%. Since the vaccine was incorporated into the provincial Expanded Program on Immunization (EPI), a total of 3.189 million doses of varicella vaccine were administered province-wide in 2023. The resulting high vaccination coverage has substantially strengthened the population-level immunity barrier against varicella [31]. The successful experience in Jiangsu Province, along with substantial domestic and international evidence, indicates that a two-dose schedule can significantly improve protective efficacy. It is therefore recommended that the varicella vaccine be included in the national childhood immunization program as soon as possible and that a two-dose schedule be adopted [31]. Each region may formulate phased targets and transitional implementation plans based on its own vaccination coverage foundation, financial capacity, and epidemiological characteristics, so as to ensure the rapid achievement of nationwide full coverage of the two-dose schedule [1,31,32].

## 5. Conclusions

This real-world study indicates that the SV-1 cell line-based varicella vaccine has a favorable safety profile among children under 6 years of age in Jiangsu Province. Reported AEFI were predominantly mild and self-limiting, with no serious adverse events identified. The increase in reporting following incorporation into the EPI likely reflects improved surveillance sensitivity rather than changes in vaccine safety. These findings support the continued implementation of the two-dose varicella vaccination strategy within routine immunization programs.

## Figures and Tables

**Table 1:** Reported AEFI after VarV (SV-1) vaccination in Jiangsu Province, 2021–2023 (per 100,000 doses).

Year	Administered Doses (×10^4^)	Common Reactions, n (per 100,000)	Abnormal Reactions, n (per 100,000)	Coincidental Events, n (per 100,000)	Total, n (per 100,000), 95% CI	
2021	4.6	17(36.96)	1(2.17)	-	18(39.12)	23.18–61.82
2022	16.73	68(40.64)	3(1.79)	-	71(42.44)	33.14–53.53
2023	99.52	525(52.75)	16(1.61)	4(0.40)	545(54.76)	50.26–59.56
Total	120.85	610(50.47)	20(1.65)	4(0.33)	634(52.46)	48.46–56.71

Reporting rates were calculated per 100,000 administered doses.

**Table 2:** Age-, sex-, dose-, and region-specific reporting rates of AEFI following VarV (SV-1) vaccination.

Characteristic	No. of AEFI	Administered Doses	Reporting Rate	95%CI	*p* Value (BH)
Sex					0.167
Female	286	578,182	49.47	43.90–55.54	
male	348	630,072	55.23	49.58–61.35	
Age(year)					<0.001 *
1	196	246,895	79.39	68.66–91.31	
2	34	82,824	41.05	28.43–57.36	
3	48	91,034	52.73	38.88–69.91	
4	213	413,478	51.51	44.83–58.92	
5	109	275,754	39.53	32.46–47.68	
6	34	98,269	34.60	23.96–48.35	
Dose number					0.015
First dose	392	687,544	57.01	51.51–62.95	
Second dose	242	520,710	46.48	40.80–52.71	
Region ^‡^					0.015
Southern Jiangsu	120	223,874	53.60	44.44–64.09	
Central Jiangsu	120	288,677	41.57	34.46–49.71	
Northern Jiangsu	394	599,950	56.63	51.18–62.51	

Reporting rates were calculated per 100,000 administered doses. * Trend across age groups was assessed using the Cochran–Armitage trend test. ^‡^ Regional classification: Southern Jiangsu includes Nanjing, Wuxi, Changzhou, Suzhou, and Zhenjiang; Central Jiangsu includes Nantong, Yangzhou, and Taizhou; Northern Jiangsu includes Xuzhou, Lianyungang, Huai’an, Yancheng, and Suqian.

**Table 3:** Multivariable mixed-effects regression analysis of factors associated with AEFI reporting rates following VarV (SV-1) vaccination.

Index	*p* Value	aIRR	95%CI
Male (vs. female)	0.216	1.12	0.94–1.33
Age 2 years (vs. 1 year)	0.001	0.50	0.33–0.74
Age 3 years (vs. 1 year)	0.010	0.63	0.44–0.89
Age 4 years (vs. 1 year)	<0.001	0.58	0.43–0.77
Age 5 years (vs. 1 year)	<0.001	0.44	0.32–0.60
Age 6 years (vs. 1 year)	<0.001	0.39	0.25–0.59
Second dose (vs. first dose)	0.468	1.10	0.86–1.40
Central Jiangsu (vs. Southern Jiangsu)	0.629	0.91	0.63–1.32
Northern Jiangsu (vs. Southern Jiangsu)	0.161	1.25	0.91–1.72

**Table 4:** Dose-specific reporting rates of adverse reactions following VarV (SV-1) vaccination, classified using MedDRA terminology.

Adverse Reactions	1st Dose, n	Reporting Rate, 95% CI	2nd Dose, n	Reporting Rate, 95% CI	Total, n	Reporting Rate, 95% CI	2nd vs. 1st, RR (95% CI)	*p* Value (BH)
All AEFI	389	56.58 (51.09–62.49)	241	46.28 (40.62–52.51)	630	52.14 (48.15–56.38)	0.82 (0.70–0.96)	0.014
All Common reactions *	377	54.83 (49.44–60.66)	233	44.75 (39.19–50.88)	610	50.49 (46.56–54.66)	0.82 (0.69–0.96)	0.027
Pyrexia	300	43.63 (38.84–48.86)	151	29.00 (24.56–34.01)	451	37.33 (33.96–40.94)	0.66 (0.55–0.81)	0.001
≥38.6 °C	144	20.94 (17.66–24.66)	68	13.06 (10.14–16.56)	212	17.55 (15.26–20.07)	0.62 (0.47–0.83)	0.006
37.1–37.5 °C	20	2.91 (1.78–4.49)	13	2.50 (1.33–4.27)	33	2.73 (1.88–3.84)	0.86 (0.43–1.73)	0.668
37.6–38.5 °C	136	19.78 (16.60–23.40)	70	13.44 (10.48–16.98)	206	17.05 (14.80–19.54)	0.68 (0.51–0.91)	0.019
Injection site induration	31	4.51 (3.06–6.40)	46	8.83 (6.47–11.78)	77	6.37 (5.03–7.96)	1.96 (1.24–3.09)	0.010
>5.0 cm	3	0.44 (0.09–1.28)	6	1.15 (0.42–2.51)	9	0.74 (0.34–1.41)	2.64 (0.66–10.56)	0.201
≤2.5 cm	16	2.33 (1.33–3.78)	23	4.42 (2.80–6.63)	39	3.23 (2.30–4.41)	1.90 (1.00–3.59)	0.071
2.6–5.0 cm	12	1.75 (0.90–3.05)	17	3.26 (1.90–5.23)	29	2.40 (1.61–3.45)	1.87 (0.89–3.92)	0.126
Injection site erythema + Injection site swelling	62	9.02 (6.91–11.56)	81	15.56 (12.35–19.33)	143	11.84 (9.97–13.94)	1.73 (1.24–2.40)	0.006
>5.0 cm	5	0.73 (0.24–1.70)	12	2.30 (1.19–4.03)	17	1.41 (0.82–2.25)	3.17 (1.12–9.00)	0.049
≤2.5 cm	28	4.07 (2.71–5.89)	24	4.61 (2.95–6.86)	52	4.30 (3.21–5.64)	1.13 (0.66–1.95)	0.668
2.6–5.0 cm	29	4.22 (2.82–6.06)	45	8.64 (6.30–11.56)	74	6.12 (4.81–7.69)	2.05 (1.28–3.27)	0.008
All Abnormal reactions	12	1.75 (0.90–3.05)	8	1.54 (0.66–3.03)	20	1.66 (1.01–2.56)	0.88 (0.36–2.15)	1.000
Allergic rash	8	1.16 (0.50–2.29)	6	1.15 (0.42–2.51)	14	1.16 (0.63–1.94)	0.99 (0.34–2.85)	1.00
Other allergic reactions	1	0.15 (0.00–0.81)	1	0.19 (0.00–1.07)	2	0.17 (0.02–0.60)	1.32 (0.08–21.11)	1.00
Urticaria	2	0.29 (0.04–1.05)	1	0.19 (0.00–1.07)	3	0.25 (0.05–0.73)	0.66 (0.06–7.28)	1.00
Viral rash	1	0.15 (0.00–0.81)	0	0.00 (0.00–0.71)	1	0.08 (0.00–0.46)	0.00 (0.00–Inf)	1.00

Reporting rates were calculated per 100,000 administered doses. * Multiple symptoms may occur in the same participant and are counted separately.

**Table 5:** Time-to-onset distribution of adverse reactions following VarV (SV-1) vaccination, classified using MedDRA terminology.

Adverse Event Category (MedDRA PT)	Day of Vaccination (%)	1–3 Days After Vaccination (%)	≥4 Days After Vaccination (%)	Total, *n*
All AEFI	66.35	32.22	1.43	630
All common reactions	66.39	32.3	1.31	610
Pyrexia	69.18	29.93	0.89	451
≥38.6 °C	71.23	28.3	0.47	212
37.1–37.5 °C	72.73	27.27	0	33
37.6–38.5 °C	66.5	32.04	1.46	206
Injection site erythema + Injection site swelling	57.34	41.96	0.7	143
>5.0 cm	29.41	70.59	0	17
≤2.5 cm	65.38	32.69	1.92	52
2.6–5.0 cm	58.11	41.89	0	74
Injection site induration	55.84	42.86	1.3	77
>5.0 cm	44.44	55.56	0	9
≤2.5 cm	56.41	41.03	2.56	39
2.6–5.0 cm	58.62	41.38	0	29
All abnormal reactions	65	30	5	20
Allergic rash	50	42.86	7.14	14
Urticaria	100	0	0	3
Other allergic reactions	100	0	0	2
Viral rash	100	0	0	1

## Data Availability

The data supporting the findings of this study were obtained from the Jiangsu Provincial Immunization Integrated Service Management Information System and the CAEFIS. Restrictions apply to the availability of these data, which were used under license for the current study and are not publicly available due to privacy and ethical considerations. Aggregated data supporting the findings of this study are available from the corresponding author upon reasonable request and with permission from the relevant authorities.

## Supplementary Materials

The following supporting information can be downloaded at https://www.mdpi.com/article/10.3390/vaccines14030200/s1, Table S1a: Distribution of reported AEFI following VarV (SV-1) vaccination among children aged <6 years in Jiangsu Province, 2021–2023. Table S1b: Annual comparisons of AEFI reporting rates following VarV (SV-1) vaccination. Table S2: City-specific AEFI reporting rates per 100,000 administered doses. Table S3: Model diagnostics and selection for count regression analyses of AEFI reporting rates. Table S4: Multivariable negative binomial regression analysis of factors associated with AEFI reporting.

## Abbreviations

The following abbreviations are used in this manuscript:

AEFI	Adverse Events Following Immunization
aIRR	Adjusted Incidence Rate Ratio
BH	Benjamini–Hochberg
CNAEFIS	Chinese National Adverse Event Following Immunization Information System
EPI	Expanded Program on Immunization
FDR	False Discovery Rate
IRR	Incidence Rate Ratio
ITS	Interrupted Time Series
MedDRA	Medical Dictionary for Regulatory Activities
NB	Negative Binomial
VarV	Varicella Vaccine
VZV	Varicella-Zoster Virus
WHO	World Health Organization
