# Supplementary material for: Safety of an SV-1 Cell Line-Based Varicella Vaccine Before and After Integration into the Expanded Program on Immunization: A Real-World Study in Jiangsu Province, China
Source: Vaccines (Basel). 2026 Feb 24;14(3):200. doi: 10.3390/vaccines14030200 (PMC13030554; doi:10.3390/vaccines14030200)
Supplement: Supplementary file 1 [file vaccines-14-00200-s001.zip › vaccines-4122526-supplementary.pdf]

Supplementary Table S1a. Distribution of reported AEFI following VarV (SV-1) vaccination among children aged <6 years in Jiangsu Province, 2021–2023

Characteristic	2021—2022			2023			Overall		
	Administered doses	AEFI cases	Reporting rate	Administered doses	AEFI cases	Reporting rate	Administered doses	AEFI cases	Reporting rate
**Sex**									
Male	11.04	48	43.50	51.99	300	57.71	63.03	348	55.22
Female	10.30	41	39.82	47.53	245	51.54	57.83	286	49.46
**Age (years)**									
1	7.89	50	63.35	16.81	146	86.87	24.70	196	79.34
2	1.48	4	26.96	6.80	30	44.11	8.28	34	41.04
3	0.51	1	19.57	8.59	47	54.69	9.10	48	52.74
4	7.79	28	35.96	33.57	185	55.11	41.36	213	51.50
5	2.38	2	8.40	25.20	107	42.47	27.58	109	39.52
6	1.28	4	31.33	8.55	30	35.08	9.83	34	34.60
**Dose number**									
1	13.53	65	48.04	55.25	327	59.19	68.78	392	56.99
2	7.80	24	30.77	44.27	218	49.24	52.07	242	46.48
**Quarter**									
1	1.48	4	26.94	57.21	286	49.99	58.68	290	49.42
2	2.48	11	44.34	27.22	156	57.31	29.70	167	56.23
3	8.67	36	41.50	11.42	70	61.31	20.09	106	52.75
4	8.69	38	43.72	3.67	33	89.86	12.36	71	57.43
**Region**									
Southern Jiangsu	5.59	25	44.71	16.80	95	56.55	22.39	120	53.59
Central Jiangsu	8.55	20	23.40	20.33	100	49.19	28.88	120	41.56
Northern Jiangsu	7.19	44	61.16	62.39	350	56.10	69.58	394	56.62
**Concomitant vaccination**									
Yes	5.06	5	9.89	13.86	12	8.66	18.92	17	8.99
No	16.28	84	51.61	85.66	533	62.22	101.94	617	60.53
**Total**	21.33	89	41.73	99.52	545	54.76	120.85	634	52.46

Note: This table provides detailed stratified data supporting the annual comparisons presented in the main text. Concomitant administration with any other vaccine on the same day; c: Including routine childhood vaccines. Reporting rate per 100,000 administered doses

Supplementary Table S1b.

Compare	RR (95%CI)	P value
2022 vs 2021	1.08 (0.64—1.93)	0.897
2023 vs 2021	1.40 (0.88—2.38)	0.182
2023 vs 2021–2022	1.31 (1.05—1.66)	0.016

Supplementary Table S2. City-specific AEFI reporting rates per 100,000 administered doses

City	AEFI cases	doses	Rate (per 100k)	95%CI	P value (BH)
Changzhou	31	61082	50.75	34.48-72.04	0.0001
Huai'an	66	128693	51.28	39.66-65.25	
Lianyungang	59	95753	61.62	46.91-79.48	
Nanjing	17	42457	40.04	23.33-64.11	

City	AEFI cases	doses	Rate (per 100k)	95%CI	P value (BH)
Nantong	49	146497	33.45	24.74-44.22	
Suzhou	14	38337	36.52	19.96-61.27	
Suqian	90	134484	66.92	53.81-82.26	
Taizhou	35	68879	50.81	35.39-70.67	
Wuxi	10	27391	36.51	17.51-67.14	
Xuzhou	138	282317	48.88	41.07-57.75	
Yancheng	41	54456	75.29	54.03-102.14	
Yangzhou	36	73301	49.11	34.40-67.99	
Zhenjiang	48	54607	87.90	64.81-116.54	

Supplementary Table S3. Model diagnostics and selection for count regression analyses of AEFI reporting rates

Model	Distribution	Random effects	Overdispersion assessment	Zero-inflation assessment	Model fit (AIC)	Conclusion
Model 1	Poisson	None	Pearson χ^2/df = 0.87	Not formally assessed	AIC = 713.65	No evidence of overdispersion based on Pearson χ^2/df; poorer fit than mixed models
Model 2	NB	None	Accommodated by dispersion parameter	Not formally assessed	AIC = 710.35	Improved fit vs Poisson (ΔAIC = 3.30)
Model 3	Poisson	City-level random intercept	DHARMa dispersion test p = 0.500	DHARMa zero-inflation test p = 0.276	AIC = 709.94	Accounts for regional heterogeneity; improved fit vs Model 1
Model 4 (Final)	NB	City-level random intercept	DHARMa dispersion test p = 0.858	DHARMa zero-inflation test p = 0.180	AIC = 709.80	Selected as primary analytical model

Notes: Overdispersion was evaluated using the Pearson chi-square statistic divided by degrees of freedom and simulation-based diagnostics. Zero inflation was assessed using DHARMa by comparing the observed number of zero counts with the expected distribution under the fitted model. Model fit was compared using Akaike information criterion (AIC).

DHARMa is a simulation-based diagnostic tool for residual diagnostics in generalized linear mixed models.

Supplementary Table S4. Multivariable NB regression analysis of factors associated with AEFI reporting (robust standard errors)

Variable	Estimate	Robust SE	P value	IRR	95% CI
Male (vs. female)	0.11	0.09	0.217	1.12	0.94-1.44
Age 2 years (vs. 1 year)	-0.71	0.21	0.001	0.49	0.33-0.75
Age 3 years (vs. 1 year)	-0.46	0.22	0.042	0.63	0.41-0.98
Age 4 years (vs. 1 year)	-0.57	0.14	<0.001	0.57	0.43-0.75
Age 5 years (vs. 1 year)	-0.84	0.14	<0.001	0.43	0.33-0.57
Age 6 years (vs. 1 year)	-0.96	0.19	<0.001	0.38	0.26-0.56
Second dose (vs. first dose)	0.10	0.11	0.351	1.10	0.90-1.36
Central Jiangsu (vs. Southern Jiangsu)	-0.15	0.16	0.361	0.86	0.63-1.18
Northern Jiangsu (vs. Southern Jiangsu)	0.17	0.14	0.206	1.19	0.91-1.55
